# Supplementary material for: Divergent creativity in humans and large language models
Source: Sci Rep. 2026 Jan 21;16:1279. doi: 10.1038/s41598-025-25157-3 (PMC12824279; doi:10.1038/s41598-025-25157-3)
Supplement: Supplementary file 1 — Supplementary Material 1 [file 41598_2025_25157_MOESM1_ESM.pdf]

Supplementary Materials for

Divergent Creativity in Humans and Large Language Models

Bellemare-Pepin et al.

*Corresponding author. Email: karim.jerbi@umontreal.ca

This PDF file includes:

Supplementary Text
Figs. S1 to S6
Table S1

Supplementary Text

Cosine similarity between haiku embeddings and the word “nature”.

The results depicted in Figure S1 demonstrate a clear trend: GPT-4-generated haikus consistently maintain a strong thematic focus on nature across all temperature settings, as evidenced by the generally high cosine similarity scores when compared to the word 'nature'. This observation suggests that GPT-4 adheres closely to the guideline of incorporating natural themes within the haikus it produces. This adherence could account for the non-significant effect of temperature variation on the Divergent Semantic Integration (DSI) scores observed in Figure 7. The relatively stable semantic alignment with the nature theme, regardless of the temperature changes, implies that GPT-4's haiku generation process is robust against such variations when it comes to maintaining thematic consistency.

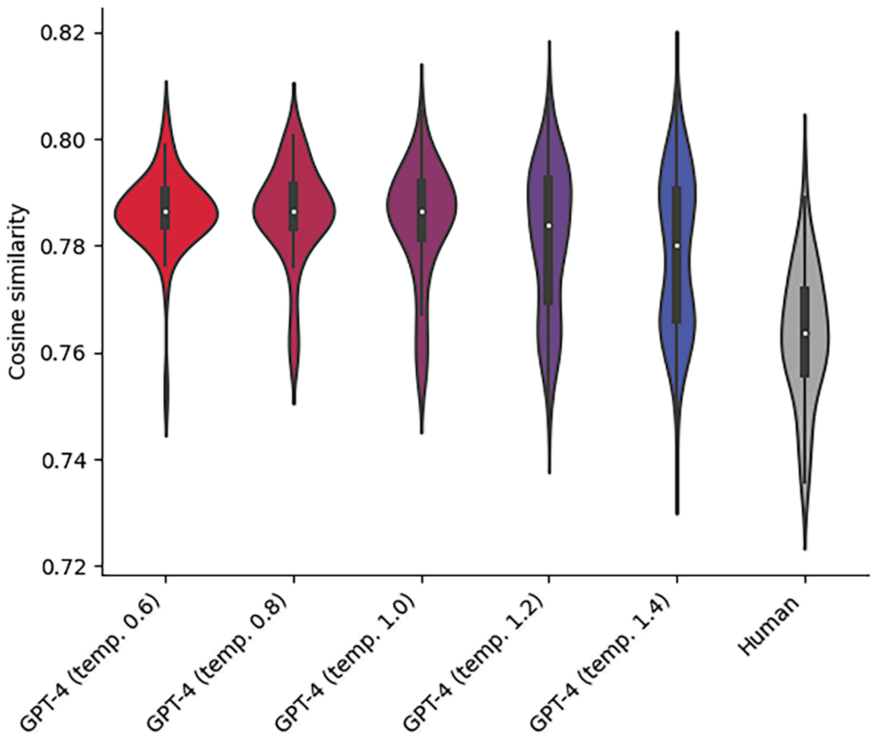

Fig. S1. Cosine similarity between haiku embeddings and the word "nature". The models represented include GPT-4 at different temperatures—0.6, 0.8, 1.0, 1.2, and 1.4—alongside a comparison with haikus authored by humans. Each plot shows the distribution of scores, with the white dot indicating the median value, the thick black bar showing the interquartile range, and the thin black lines denoting the overall range of data. The spread of each plot suggests the degree to which the haikus are semantically related to 'nature',

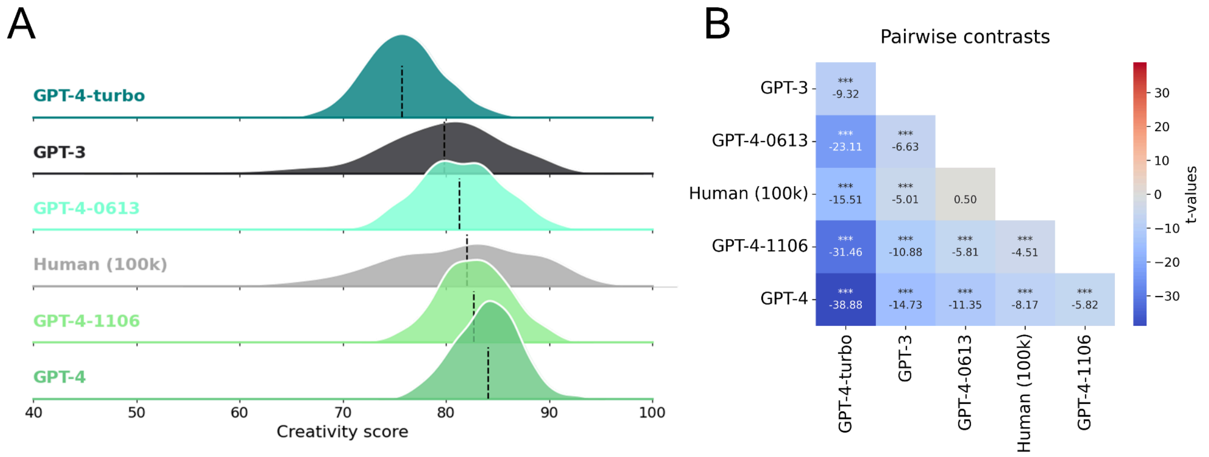

Fig. S2. Comparing GPT-4 models and humans on the Divergent Association Task (DAT). The models represented include GPT-3.5-turbo, GPT-4-0314, GPT-4-0613, GPT-4-1106 and GPT-4-turbo. **(A)** Mean DAT score and 95% confidence intervals. **(B)** Heatmap of all contrasts using independent t-tests, sorted by their correlation with the highest performing model, GPT-4-0314.

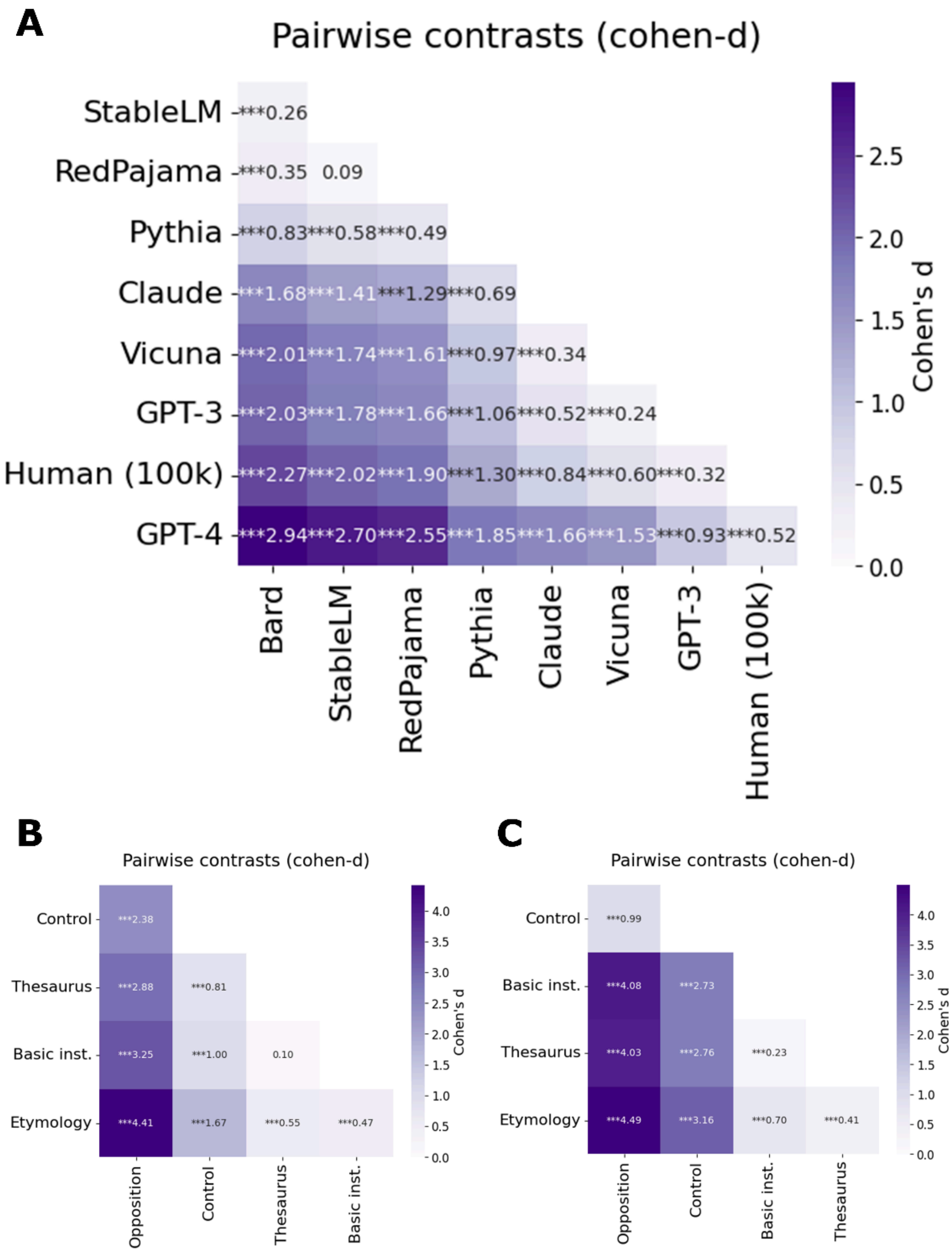

Fig. S3. Effect size of the main results from Figures 1 and 4. (A) Heatmap illustrating *Cohen's d* for all pairwise comparisons of DAT scores between LLMs and human responses. **(B)** Heatmap showing *Cohen's d* for comparisons of DAT scores across different GPT-3.5 strategies. **(C)** Heatmap depicting *Cohen's d* for comparisons of DAT scores across different GPT-4 strategies.

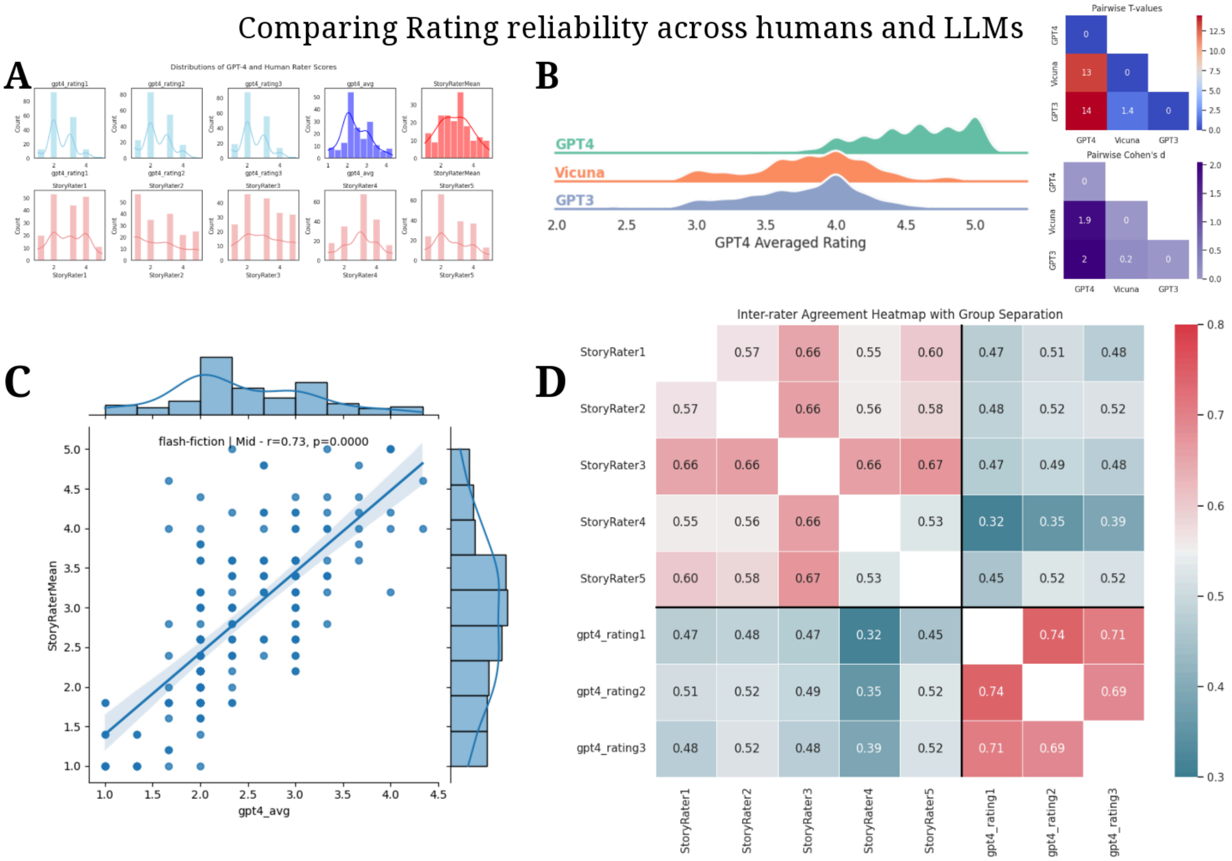

Fig. S4. Assessment of inter-rater reliability across humans and GPT-4. (A) Histograms showing the distribution of creativity scores given on an ordinal scale between 1 and 5 representing judgements of overall perceived creativity. **(B)** Distribution of GPT-4 averaged creativity judgements (ratings) show a significant difference between GPT-4 and Vicuna/GPT-3 [add stats]**(C)** A significant correlation was found between averaged judgements in GPT-4 and human raters [add stats] **(D)** Heatmap showing the inter-rater agreement of these judgements. There was a significantly lower concordance between human and GPT-4 raters (M = 0.47, SD = 0.06) compared to within human judgments (M = 0.61, SD = 0.05), $t(28) = 5.86$, $p < .001$, d = 2.54.

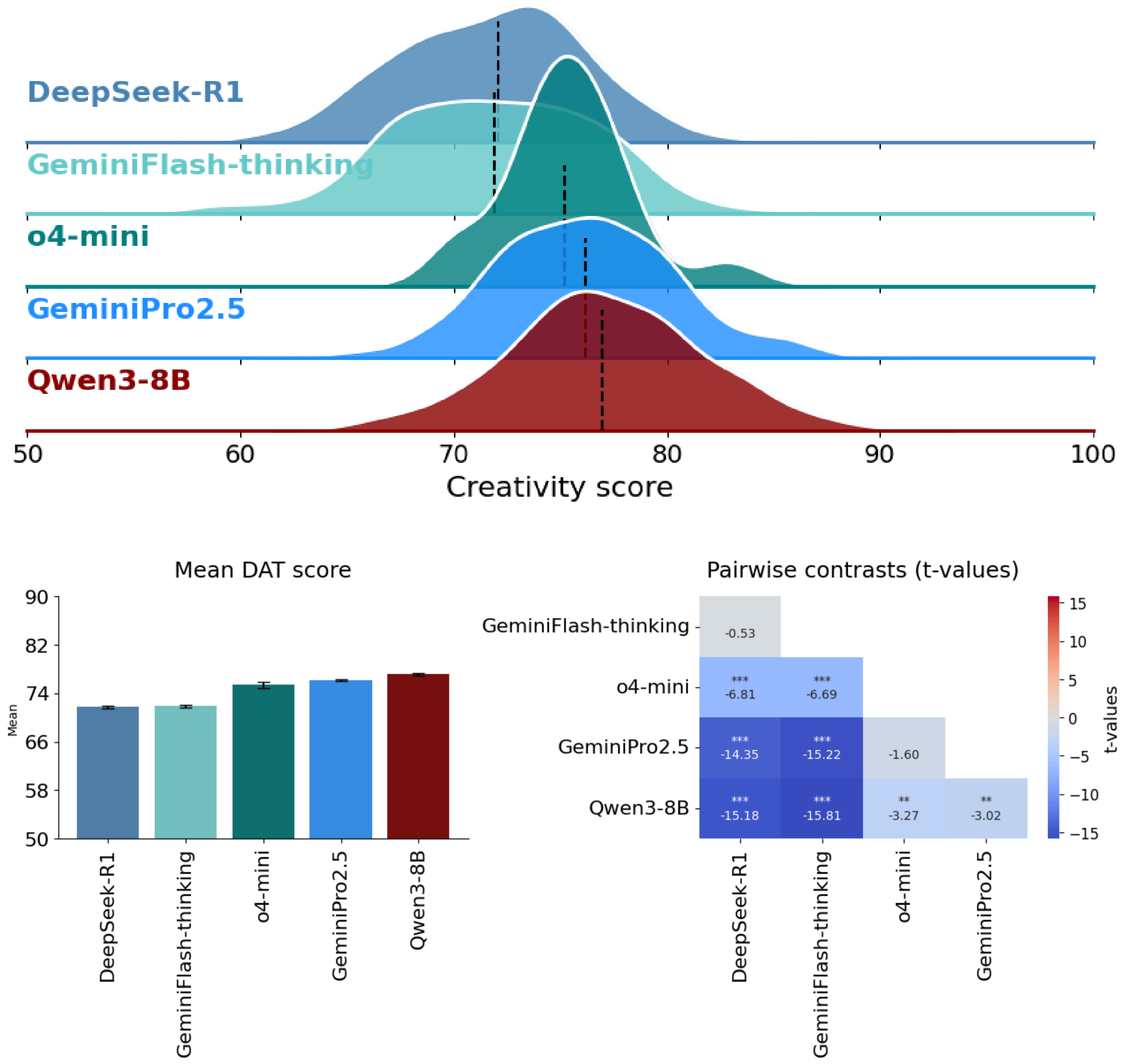

Fig S5. Comparing "Reasoning" models on the Divergent Association Task (DAT).

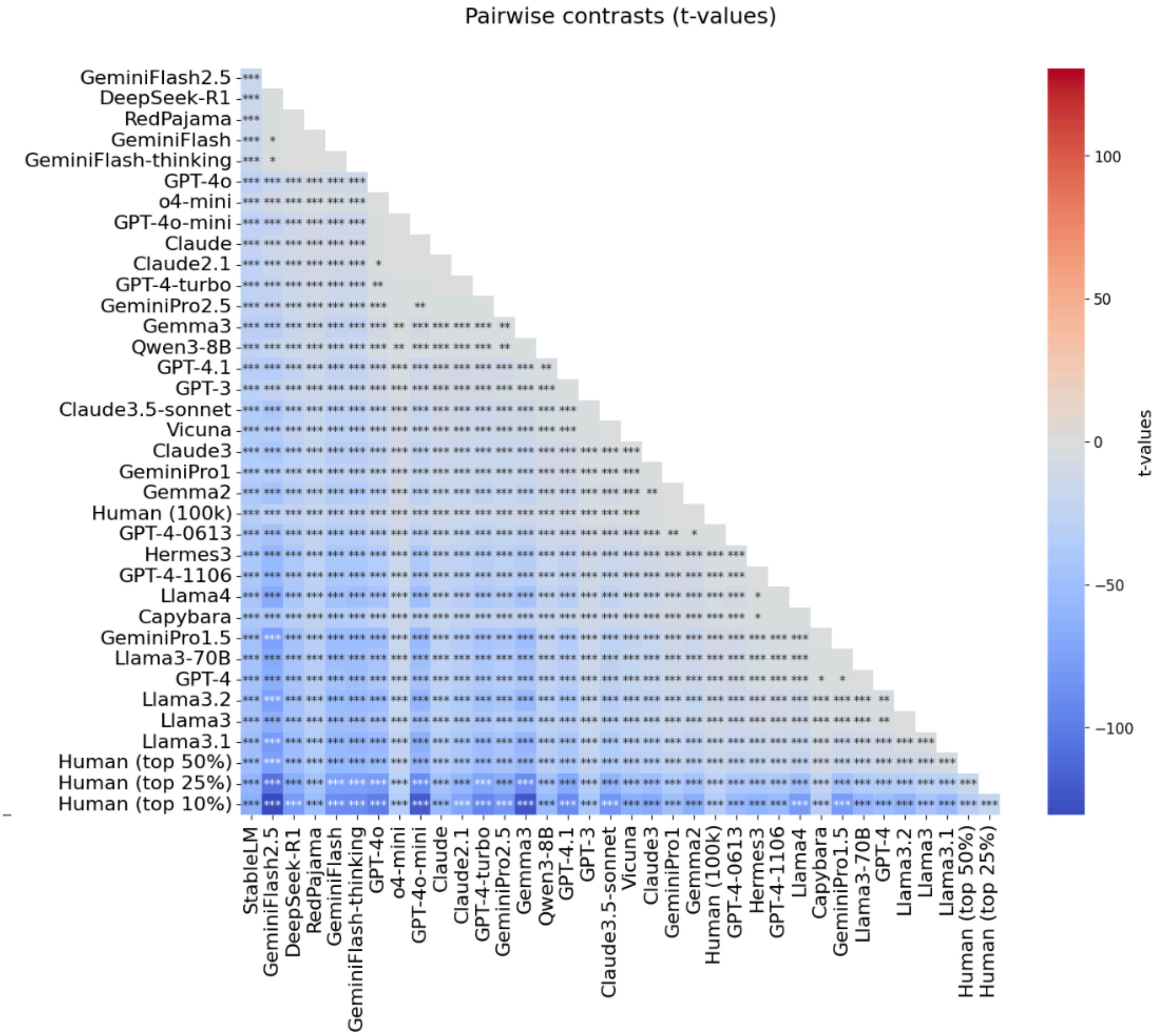

Fig S6. Pairwise independent t-tests (t-values) comparing mean creativity scores across large language models (LLMs) and human samples on the Divergent Association Task (DAT). Models and human benchmarks (full distribution, top 50%, 25%, and 10%) are ranked from lowest to highest mean score. Each cell displays the t-value for the contrast between the row and column models. Statistical significance is indicated by asterisks (* $p < .05$, ** $p < .01$, *** $p < .001$) after false discovery rate (FDR) correction for multiple comparisons.

Model name	Organization	Model ID	Model size	Fine-tuning	Temp range/ default	License	Source	Year of release
Capybara	LAION	laion/oasst-sft-7b-llama-3.1	7B	RLHF	0.7	Apache 2.0	Hugging Face	2023
Claude	Anthropic	claude-instant-1.2	unknown	RLAIF	0-1 / 0.7	Paid access	Anthropic API	2023
Claude2.1	Anthropic	claude-2.1	unknown	RLAIF	0-1 / 0.7	Paid access	Anthropic API	2023
Claude3	Anthropic	claude-3-opus-20240229	unknown	RLAIF	0-1 / 0.7	Paid access	Anthropic API	2024
Claude3.5-sonnet	Anthropic	claude-3-5-sonnet-20240620	unknown	RLAIF	0-1 / 0.7	Paid access	Anthropic API	2024
DeepSeek-R1	DeepSeek AI	deepseek-coder-6.7b-instruct	6.7B	RLHF	0.7	MIT	Hugging Face	2024
Gemma2	Google	gemma-2-9b-it	9B	Instruction Tuning	0.7	Gemma License	Google AI	2024
Gemma3	Google	gemma3:27b	27B	RLHF	n.a.	Gemma 3 License	ollama	2025
GeminiFlash	Google	gemini-1.5-flash-latest	1.5M tokens	Instruction Tuning	0-2 / 1.0	Paid access	Google AI Platform	2024
GeminiFlash-thinking	Google	gemini-2.0-flash-thinking-exp-1219	Undisclosed	Undisclosed	n.a.	Paid access	Google AI	2024
GeminiPro1	Google	gemini-1.0-pro	unknown	Instruction Tuning	0-1 / 0.9	Paid access	Google AI Platform	2023
GeminiPro1.5	Google	gemini-1.5-pro-latest	1M tokens	Instruction Tuning	0-2 / 1.0	Paid access	Google AI Platform	2024
GeminiPro2.5	Google	gemini-2.5-pro-preview-05-06	Undisclosed	Undisclosed	n.a.	Paid access	Google AI	2025
GeminiFlash2.5	Google	gemini-2.5-flash	Undisclosed	Undisclosed	n.a.	Paid access	Google AI	2025
GPT-3	OpenAI	gpt-3.5-turbo	175B	RLHF	0-2 / 1	Paid access	OpenAI API	2022
GPT-4	OpenAI	gpt-4	unknown	RLHF	0-2 / 1	Paid access	OpenAI API	2023
GPT-4-0613	OpenAI	gpt-4-0613	unknown	RLHF	0-2 / 1	Paid access	OpenAI API	2023
GPT-4-1106	OpenAI	gpt-4-1106-preview	128k tokens	RLHF	0-2 / 1	Paid access	OpenAI API	2023
GPT-4-turbo	OpenAI	gpt-4-turbo	128k tokens	RLHF	0-2 / 1	Paid access	OpenAI API	2024
GPT-4o	OpenAI	gpt-4o	unknown	RLHF	0-2 / 1	Paid access	OpenAI API	2024
GPT-4o-mini	OpenAI	gpt-4o-mini	unknown	RLHF	0-2 / 1	Paid access	OpenAI API	2024
GPT-4.1	OpenAI	gpt-4.1-2025-04-16	Undisclosed	Undisclosed	0.0 - 2.0 /1.0	Paid access	OpenAI API	2025
Hermes3	Nous Research	Nous-Hermes-2-Yi-34B	34B	Instruction Tuning	0.7	Apache 2.0	Hugging Face	2023
Llama3	Meta	meta-llama/Llama-3-8B-Instruct	8B	RLHF	1	Llama 3 License	Hugging Face	2024
Llama3-70B	Meta	meta-llama/Llama-3-70B-Instruct	70B	RLHF	1	Llama 3 License	Hugging Face	2024
Llama3.1	Meta	meta-llama/Llama-3.1-8B-Instruct	8B	RLHF	1	Llama 3.1 License	Hugging Face	2024
Llama3.2	Meta	llama3.2:3b	3B	RLHF	1	Llama 3.2 Community License Agreement	ollama	2024
Llama4	Meta	llama4:latest	16x17B	RLHF	1	Llama 4 Community License Agreement	ollama	2025
o4-mini	OpenAI	o4-mini-2025-04-16	unknown	RLHF	1	Paid access	OpenAI API	2025
Qwen3-8B	Alibaba Cloud	Qwen1.5-7B-Chat	7B	RLHF	n.a.	Apache 2.0	Hugging Face	2024
RedPajama	Together	RedPajama-INCITE-Chat-7B-v0.1	7B	RLHF	0-1 / 0.7	Apache 2.0	Hugging Face	2023
StableLM	Stability AI	stablelm-2-zephyr-1.6b	1.6B	RLHF	n.a.	Stability AI License	Hugging Face	2024
Vicuna	LMSYS	lmsys/vicuna-7b-v1.5	7B	RLHF	0.7	Apache 2.0	Hugging Face	2023

Table S1. Full list of model specifications.
